# Red blood cell distribution width to platelet ratio substantiates preoperative survival prediction in patients with newly-diagnosed glioblastoma

**DOI:** 10.1007/s11060-021-03817-4

**Published:** 2021-08-04

**Authors:** Matthias Schneider, Niklas Schäfer, Stefanos Apallas, Anna-Laura Potthoff, Christian Bode, Erdem Güresir, Muriel Heimann, Felix Lehmann, Elisa Scharnböck, Christina Schaub, Hartmut Vatter, Ulrich Herrlinger, Patrick Schuss

**Affiliations:** 1 Department of Neurosurgery, University Hospital Bonn, Venusberg-Campus 1, 53127 Bonn, Germany; 2 Division of Clinical Neuro-Oncology, Department of Neurology, University Hospital Bonn, Bonn, Germany; 3 Department of Anesthesiology and Intensive Care Medicine, University Hospital Bonn, Bonn, Germany

**Keywords:** Inflammation, Glioblastoma, Red blood cell distribution width, Platelet

## Abstract

**Object:**

The conception of individual patient-adjusted treatment strategies is constantly emerging in the field of neuro-oncology. Systemic laboratory markers may allow insights into individual needs and estimated treatment benefit at an earliest possible stage. Therefore, the present study was aimed at analyzing the prognostic significance of preoperative routine laboratory values in patients with newly-diagnosed glioblastoma.

**Methods:**

Between 2014 and 2019, 257 patients were surgically treated for newly-diagnosed glioblastoma at the Neuro-Oncology Center of the University Hospital Bonn. Preoperative routine laboratory values including red blood cell distribution width (RDW) and platelet count were reviewed. RDW to platelet count ratio (RPR) was calculated and correlated to overall survival (OS) rates.

**Results:**

Median preoperative RPR was 0.053 (IQR 0.044–0.062). The receiver operating characteristic (ROC) curve indicated an optimal cut-off value for RPR to be 0.05 (AUC 0.62; p = 0.002, 95% CI 0.544–0.685). 101 patients (39%) presented with a preoperative RPR < 0.05, whereas 156 patients (61%) had a RPR ≥ 0.05. Patients with preoperative RPR < 0.05 exhibited a median OS of 20 months (95% CI 17.9–22.1), which was significantly higher compared to a median OS of 13 months (95% CI 10.9–15.1) in patients with preoperative RPR ≥ 0.05 (p < 0.001).

**Conclusions:**

The present study suggests the RPR to constitute a novel prognostic inflammatory marker for glioblastoma patients in the course of preoperative routine laboratory examinations and might contribute to a personalized medicine approach.

## Introduction

Despite many efforts, glioblastoma is still associated with a dismal prognosis and poses an interdisciplinary challenge to patient-centered collaboration for its treatment [1]. Therefore, an early and reliable prognostication of a patient's survival, preferably prior to surgery, is of utmost relevance [2]. The emphasis herein is not to withhold potential treatment options, but rather to tailor these therapeutic strategies to the individual needs of the patient or to their estimated benefit [3]. In addition to various efforts in identification of clinical risk features, laboratory markers represent a clearly objectifiable target of academic endeavors for early detection of patients at risk. Laboratory markers have also been used in other cancers to assess disease progression and/or survival in clinical practice [4, 5]. Pierscianek et al. previously identified several laboratory parameters that might predict survival of patients with glioblastoma [6, 7]. Here, among the most promising markers, inflammatory parameters are consistently found to be associated with survival in glioblastoma patients. Inflammatory response has long been associated with assessment of cancer development, progression and prognosis. Nevertheless, non-specific laboratory values such as c-reactive protein (CRP), fibrinogen or white blood cell (WBC) count often represent imprecise parameters that may be influenced by various conditions. For example, preoperative administration of glucocorticoids is common practice in glioblastoma patients and may result in a cortison-induced leukocytosis associated with a poor prognosis [8]. However, more specific parameters (including differential blood work) are again often not part of the routinely collected preoperative laboratory values, making them unsuitable for widespread use.

To date, the prognostic value of red blood cell distribution width (RDW) has been noted for various diseases, most recently including various cancers [5, 9–11]. There are increasing reports emphasizing the inflammatory and also prognostic significance of the RDW to platelet count ratio (RPR) [12, 13]. Both RDW and platelet count belong to routine preoperative laboratory parameters, the former from a historical point of view to help classify eventual anemia. To our knowledge, the ratio calculated therefrom (RPR) has not yet been studied in its association with overall survival in glioblastoma patients.

Therefore, we analyzed patients with newly-diagnosed glioblastoma who had undergone surgery in our neuro-oncology center regarding the prognostic significance of preoperative routine laboratory values with a special focus on the RPR.

## Materials and methods

### Patients

All patients with newly diagnosed glioblastoma treated surgically at the authors' institution between 2014 and 2019 were entered into a computerized database (SPSS, version 25, IBM Corp., Armonk, NY). Approval was obtained from the institutional ethics committee for conducting the present study. To homogenize the patient cohort, only patients who underwent surgical resection were included, thus excluding patients with biopsy alone or no surgical intervention at all. In addition, only patients for whom the information listed below remained complete were included in further analysis.

To focus on the information available preoperatively, patient records were retrospectively reviewed for information such as patient characteristics, radiological features, results of preoperative laboratory tests, as well as functional neurological status at admission and during the course of treatment for further analysis. Thereby, the Karnofsky Performance Score (KPS) was used to grade patients according to their neurological functional status preoperatively, postoperatively, and during follow-up. In this regard, a KPS ≥ 70 was defined as favorable outcome. Established prognostic factors, such as the extent of surgical resection, postoperative prolonged mechanical ventilation (PMV) and/or additional molecular-pathological features (e.g., MGMT status) were discarded from additional multivariable analysis to focus solely on preoperatively available information. As previously reported, treatment decisions were reached in an interdisciplinary and consensus manner at the patient's initial presentation and during follow-up via the institutional tumor board meetings [14, 15].

Regarding laboratory analyses, values such as serum CRP (CRP < 3 g/dl versus [vs.] ≥ 3 g/dl), white blood cell (WBC) count (≤ 12 G/l vs. > 12 G/l), platelet count, RDW (≤ 14% vs. > 14%), and hemoglobin (Hb) were obtained in the routine preoperative laboratory tests and dichotomized according to laboratory-defined normal-range values and/or previous experience [14, 16–18]. Anemia was defined sex-specific according to the World Health Organization (WHO) classification (Hb < 12 g/dl for women and Hb < 13 g/dl for men) [19].

Overall survival (OS) was measured from the day of glioblastoma surgery until death or last observation. All parameters were compared in relation to OS.

### Statistics

Data analysis was accomplished using the SPSS computer software package (version 25, IBM Corp., Armonk, NY). Unpaired categorical and binary variables were analyzed in contingency tables using Fisher's exact test. For comparison of continuous variables, the Mann–Whitney U test was chosen since the data were mostly not normally distributed. To assess the discriminatory ability of the RPR in prognostic prediction, a receiver operating characteristic (ROC) curve was constructed within the studied patient population and the area under the curve (AUC) was calculated. A similar approach was taken for the optimal cut-off value for platelet count, as available literature provides sparse or contradictory information in this regard [20]. The optimal cut-off value for the corresponding values was determined from the curve over the given sensitivity and specificity. OS was analyzed with the Kaplan–Meier method using the Gehan-Breslow-Wilcoxon test. Results with p < 0.05 were considered statistically significant.

In addition, a multivariable logistic Cox regression model was constructed in order to identify independent preoperative predictors of OS in patients with glioblastoma undergoing surgical resection.

## Results

### Patient characteristics

During the period from 2014 to 2019, a total of 257 patients underwent surgical treatment for newly diagnosed glioblastoma at the Neuro-Oncology Center of the University Hospital Bonn and have been included in further analysis. Median age amounted to 64 years (interquartile range [IQR] 53–72 years). Patients reviewed for surgically treated glioblastoma in this study presented preoperatively with a median KPS of 90 (IQR 80–90). Gross-total resection (GTR) in the sense of complete removal of all contrast-enhancing tumor tissue was achieved in 176 patients (69%), subtotal resection (STR) in 81 patients (31%). Median OS (mOS) of the entire patient cohort with surgically treated glioblastoma was 16 months (95% CI 14.2–17.8).

### Preoperative laboratory panel

The median preoperative CRP level was 0.9 mg/l (IQR 0.4–2.7). Overall, 199 patients (77%) demonstrated a CRP < 3 mg/l preoperatively, while 58 patients (23%) had a CRP ≥ 3 mg/l. Patients with a preoperative CRP < 3 mg/L achieved a mOS of 16 months (95% CI 14.1–17.9) compared to a mOS of 12 months (95% CI 9.2–14.8) in patients with a preoperative CRP ≥ 3 mg/l (p = 0.121).

Median WBC count in patients with glioblastoma requiring surgery was 10.5 G/l (IQR 7.3–15.2). Overall, 154 patients (60%) demonstrated a WBC count ≤ 12 G/l preoperatively, while 103 patients (40%) had a WBC count > 12 G/l. In patients with a preoperative WBC count ≤ 12 G/l had mOS was 16 months (95% CI 14.1–17.9) compared to a mOS of 15 months (95% CI 10.9–19.1) in patients with a preoperative WBC count > 12 G/l (p = 0.655).

**Fig. 1:**
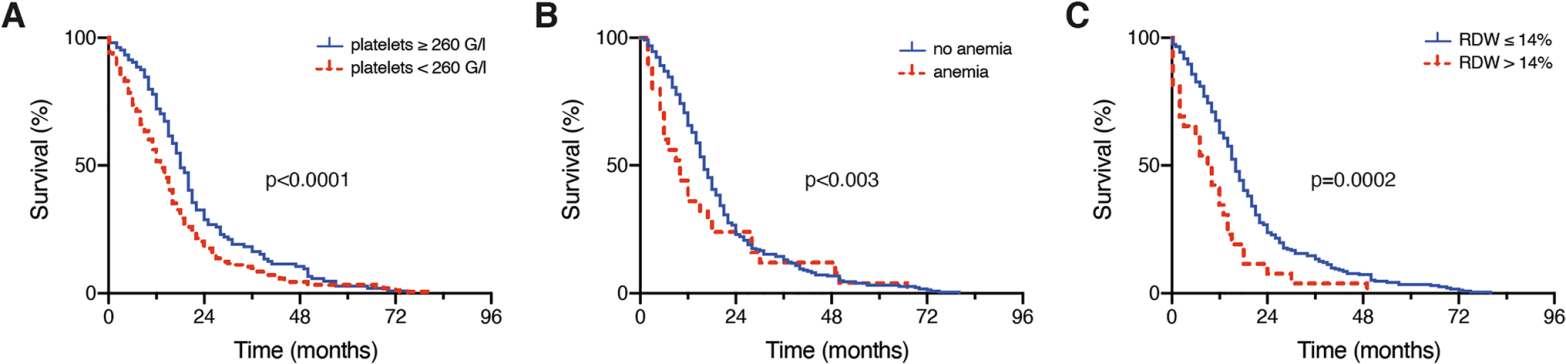
Kaplan–Meier survival curves depict the influence of **A** preoperative platelet count (≥ 260 G/l, < 260 G/l), **B** preoperative presence of anemia and **C** preoperative RDW (≤ 14%, > 14%) on OS in patients with newly-diagnosed glioblastoma. *RDW* red blood cell distribution width, *OS* overall survival

Patients with glioblastoma requiring surgery had a median platelet count of 247 G/l (IQR 214–294) preoperatively. The ROC curve indicated the optimal cut-off value for platelet count in the present study cohort to be 260 G/l (AUC 0.60; p = 0.006, 95% CI 0.532–0.672) with a sensitivity of 72% and a specificity of 52%. Given this, 153 patients (60%) presented with a platelet count ≤ 260 G/l preoperatively, whereas 104 patients (40%) had a platelet count > 260 G/l. In patients with a preoperative platelet count > 260 G/l survival was prolonged (mOS 20 months, 95% CI 17.8–22.2) compared to patients with a preoperative platelet count ≤ 260 G/l (mOS 13 months, 95% CI 10.8–15.2; p < 0.0001, Fig. 1A).

Median Hb level at the time of hospital admission prior to scheduled surgery for glioblastoma was 14.7 g/dl (IQR 13.6–15.6). A total of 27 patients (11%) with glioblastoma requiring surgery exhibited preoperative anemia (after sex-specific adjustments). Patients with preoperative anemia achieved a significantly lower mOS (9 months, 95% CI 3.9–14.1) compared to the mOS of patients without preoperative prominent anemic laboratory findings (16 months, 95% CI 14.2–17.8; p = 0.003, Fig. 1B).

Median RDW in patients with glioblastoma requiring surgery was 12.9% (IQR 12.3–13.3). Overall, 231 patients (90%) presented with a preoperative RDW ≤ 14%, while 26 patients (10%) had a preoperative RDW > 14%. Patients with a preoperative RDW ≤ 14% exhibited a higher mOS (17 months, 95% CI 15.2–18.7) compared to the mOS of patients with preoperative RDW > 14% (9 months, 95% CI 5.0–12.9; p < 0.001; Fig. 1C).

### Influence of RPR on overall survival

Median RPR was 0.053 in patients with glioblastoma prior surgery (IQR 0.044–0.062). The ROC curve indicated the optimal cut-off value for RPR to be 0.05 (AUC 0.62; p = 0.002, 95% CI 0.544–0.685) with a sensitivity of 71% and a specificity of 52%. Given this, 101 patients (39%) presented with a preoperative RPR < 0.05, whereas 156 patients (61%) had a RPR ≥ 0.05. Table 1 depicts the distribution of known prognostic parameters in the respective groups after the patient cohort was divided based on the determined RPR cut-off value.

**Table 1:** Distribution of known prognostic parameters

	RPR < 0.05 (n = 101)	RPR ≥ 0.05 (n = 156)	
Median age (IQR)	58 (51–68)	66 (57–73)	p < 0.0001
Preoperative KPS ≥ 70	95 (94%)	149 (96%)	p = 0.8
GTR	75 (74%)	101 (65%)	p = 0.1
MGMT non-methylated	52 (52%)*	90 (59%)**	p = 0.3
Postoperative PMV (> 24 h)	4 (4%)	14 (9%)	p = 0.1

∗Information missing in 1 patient (1%)

∗∗Information missing in 4 patients (3%)

**Fig. 2:**
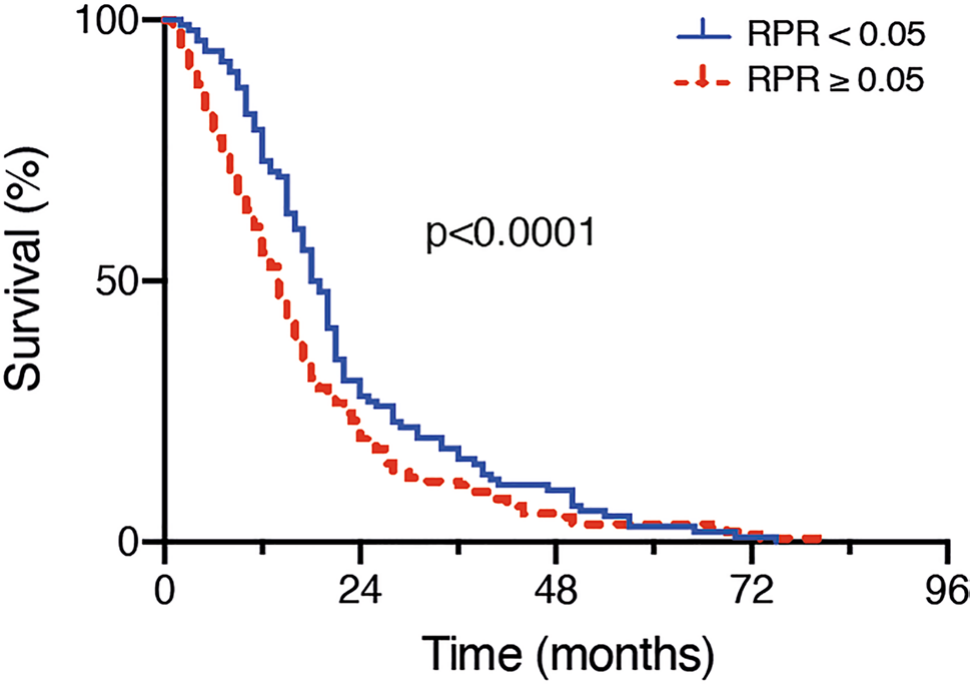
Kaplan–Meier survival curves dependent on the preoperative RPR cut-off value of 0.05 in patients with newly-diagnosed glioblastoma. *RPR* red blood cell distribution width to platelet count ratio

In glioblastoma patients with preoperative RPR < 0.05 median OS was 20 months (95% CI 17.9–22.1), which was significantly higher compared to a median OS of 13 months (95% CI 10.9–15.1) in patients with preoperative RPR ≥ 0.05 (p < 0.001; Fig. 2).

### Multivariable analysis

An additional multivariable survival analysis was performed to identify preoperatively-collectable independent predictors of OS available in patients with glioblastoma. The multivariable analysis identified the known variable "age ≥ 65 years" (p < 0.0001, HR 2.2, 95% CI 1.6–2.9) together with the laboratory value ratio "RPR > 0.05" (p = 0.037, HR 1.4, 95% CI 1.1–1.8) as significant and independent preoperatively amenable predictors of poor OS in patients with glioblastoma requiring surgery.

## Discussion

The present study identifies the red blood cell distribution width (RDW)-to-platelet ratio (RPR) as a laboratory value obtainable preoperatively which might be established as an independent predictor of OS in patients with glioblastoma requiring surgery.

Due to their aggressiveness and the persistently grim overall prognosis (despite all efforts), glioblastomas are an enormous burden for the affected patient, the family members/caregivers and also the treating physicians [21, 22]. An initial landmark assessment of the risk/benefit ratio is desirable prior to any potential treatment offer [15]. Various prognostic factors such as age, initial neurological status (KPS), extent of resection, MGMT status, and postoperative intensive care complications (PMV) have been established or suggested for this assessment [14, 15, 23]. However, a large part of this refers to the postoperative/biopsy situation, when information on resection extent, molecular pathology, and/or complicative course becomes available [24]. In the search for risk factors for overall survival that can be elected preoperatively if possible, laboratory indicators also repeatedly come to the fore [4, 7]. Here, special attention is given to inflammatory markers. Many authors have previously highlighted a pivotal association between chronic inflammatory processes and the development/progression of tumors [4, 5, 25, 26]. Indeed, it is well known that the carcinogenesis process implies a complex interaction of inflammatory cells in a specific local microenvironment in the tumor site [27–29]. These inflammatory processes take a decisive position in the stimulation of tumor growth, angiogenesis and further tumor infiltration [28]. Given that glioblastoma as such does not tend to metastasize outside the central nervous system, the assumption of a fundamental correlation and interaction between the systemic and local inflammation in the case of glioblastoma is not entirely comprehensible. There is growing evidence that exosomes among others may play an important role within these systemic inflammatory responses [30]. Along these lines, glioblastoma-derived exosomes have been shown to convert M1 macrophages into tumor-associated macrophages (TAMs) by transcriptional cellular reprogramming. These reprogrammed TAMs in turn produce exosomes that exert systemic inflammatory responses therefore highliting exosomes as effectors of key inflammation-related players [31]. Nevertheless, these experimental insights and also experience from other cancer entities have led to the identification of some hematologic prognostic parameters that, in the context of a low-cost and routinely collected preoperative blood test, could lead to better predictability and thus better management by focusing on patients at risk [26].

RDW has been established as a biomarker for predicting mortality in various diseases [32–35]. Nevertheless, the underlying pathophysiological mechanism remains mostly unexplained and thus—as often—statistical uncertainties are discussed. Nevertheless, the predictive ability of RDW, for example in cardiac diseases, remains undisputed despite multiple attempts [36]. RDW has also been found to be a prognosticator in a wide variety of cancers [37–39]. Where RDW has already gained prognostic value in predicting overall survival in patients with glioblastoma [18], the ratio formed from it together with platelet count (RPR) is experiencing increasing interest in other diseases [12, 13, 40, 41]. Platelet count as such is of unsteady predictive value. This is due to the fact that the normal distribution of platelet counts contains a range and not a cut-off value, so that the individually adjusted cut-off values in the individual studies are not comparable. In glioblastoma, an increase in platelet count was previously assumed to be an unfavorable prognostic feature [41]. This fits with the experience in most solid tumors that the elevated platelet count plays a critical role in progression and metastasis [42–44]. Presumed mechanisms include protecting circulating tumor cells from attacking the host's immune system as well as supporting proliferation of tumor cells [45]. However, glioblastoma differs from other solid tumors in the low to nearly absent frequency of systemically circulating tumor cells, which makes this argumentation of the observation regarding platelet count rather challenging to substantiate [46, 47]. In the present study, patients with an increased preoperative platelet count exhibited better OS in contrast to patients with a decreased count. Furthermore, the present study reveals a survival benefit for the group a low ratio of RDW to platelets, the RPR, which uses absolute numbers and thus can be derived from any routine blood test. RPR was first introduced to predict hepatic fibrosis in hepatitis [48]. Although the pathophysiological background of the inflammation marker RPR remains unclear, its elevation serves the probability of an increased RDW and a decreased platelet count. The advantage with regard to the platelet count is certainly the use of the count without prior cut-off limitation. However, in the present patient cohort, nonspecific inflammation markers such as CRP and WBC count do not seem to be suitable for preoperative prognostic assessment. This may be due to the non-specificity of these markers as well as to the possible adjustments prior to (semi-)elective surgery: in addition to the preoperative clarification of potential infection, the administration of glucocorticoids in glioblastoma patients should also be mentioned here.

Although the precise mechanism remains unclear, the present study indicates for the first time a potential prognostic value of the inflammatory marker RPR in glioblastoma patients.

### Limitations

In addition to the retrospective design, the present work has further inherent limitations. These include the fact that the recorded and analyzed inflammatory laboratory values were preoperative in nature. In the case of (semi-)elective surgery and retrospective data collection, there is a risk that the actual deviation of the recorded laboratory values has already been corrected by preoperative measures. Nevertheless, this work considers the RPR as a preoperatively-collectable prognostic marker in glioblastoma patients for the first time and should thereby aid in accelerating further study efforts to validate this correlation in other neurooncological centers.

## Conclusions

The present study suggests the RPR to constitute a novel prognostic inflammatory marker for glioblastoma patients in the course of preoperative routine laboratory examinations and might contribute to a personalized medicine approach.

## Data Availability

Restrictions apply to the availability of these data due to privacy restrictions.
